# Informing future randomized controlled trials of amantadine hydrochloride in neurocritical care and post-neurocritical care stroke patients through a retrospective study

**DOI:** 10.1186/s12883-024-03854-2

**Published:** 2024-09-11

**Authors:** Enzo G. Plaitano, Rebecca A. Scharf, Pakinam E. Aboutaleb, Andrea L. Glennon, Emiliya Melkumova, Deborah M. Green-LaRoche

**Affiliations:** 1 Department of Neurology, Tufts Medical Center, 800 Washington Street #314, Boston, MA 02111 USA; 2 Undergraduate Program in Neuroscience, Boston University, Boston, MA USA; 3 Department of Pharmacy, Tufts Medical Center, Boston, MA USA

**Keywords:** Amantadine hydrochloride, Ischemic stroke, Hemorrhagic stroke, Critical care, Hospitalization, Retrospective studies

## Abstract

**Background:**

Amantadine hydrochloride has been increasingly prescribed as a neurostimulant for neurocritical care stroke patients to promote wakefulness during inpatient recovery. However, a lack of guidelines makes it difficult to decide who may benefit from this pharmacotherapy and when amantadine should be initiated during the hospital stay. This study aims to determine some factors that may be associated with favorable response to amantadine to inform future randomized controlled trials of amantadine in critical care or post-critical care stroke patients.

**Methods:**

Retrospective chart review for this study included neurocritical care and post-neurocritical care patients with acute ischemic or hemorrhagic stroke who were started on amantadine (*N* = 34) in the years 2016–2019. Patients were labeled as either responders or nonresponders of amantadine within 9 days of initiation using novel amantadine scoring criteria utilized and published in Neurocritical Care in the year 2021, which included spontaneous wakefulness and Glasgow Coma Scale (GCS). Amantadine response status and predictive variables were analyzed using nonparametric tests and adjusted multivariable regression models.

**Results:**

There were large but nonsignificant variations in the median total milligrams of amantadine received in the first 9 days (IQR = 700-1,450 mg, *p* = 0.727). GCS on the day before amantadine initiation was significantly higher for responders (median = 12, IQR = 9–14) than nonresponders (median = 9, IQR = 8–10, *p* = 0.009). Favorable responder status was significantly associated with initiation in the critical care unit versus the step-down unit or the general medical/surgical floor [𝛃=1.02, 95% CI (0.10, 1.93), *p* = 0.031], but there was no significant associations with hospital day number started [𝛃=-0.003, 95% CI (-0.02, 0.02), *p* = 0.772].

**Conclusions:**

Future randomized controlled trials of amantadine in hospitalized stroke patients should possibly consider examining dose-dependent relationships to establish stroke-specific dosing guidelines, minimum GCS threshold for which amantadine is efficacious, and the impact of patients’ determined *level of acuity* on clinical outcomes instead of solely examining the impact of earlier amantadine initiation by hospital day number. Future research with larger sample sizes is needed to further examine these relationships and inform future clinical trials.

## Background

Stroke is the fourth leading cause of death and the number one cause of long-term disability among adults in the United States [1]. The annual increase in functional disability triples after stroke is factored into the analysis, with approximately 5% of the US population citing stroke as their cause of disability [2, 3]. Additional meta-analysis shows a significant negative trend between cognitive function and stroke, suggesting that many survivors suffer from cognitive decline [4].

The number of patients admitted to critical care units for post-stroke management and complications is growing [5]. Nearly 40% of adult patients who spend extended time in critical care units experience decreased motor abilities upon recovery, and 30–80% experience long-term cognitive deficits [6, 7]. These patients are frequently bedridden or comatose and have trouble participating in inpatient therapy, which contributes to worse long-term outcomes [7].

In response, neurocritical care physicians have turned to pharmacological interventions to mediate recovery during acute rehabilitation in the critical care units. The pharmaceutical drug amantadine hydrochloride, or amantadine, for short, is a synthetic tricyclic amine that was originally prescribed as an influenza prophylactic agent due to its antiviral properties [8]. Amantadine is believed to increase wakefulness through increasing dopamine (DA) synthesis and blocking reuptake, therefore increasing the amount of DA available to bind to receptors on the postsynaptic neuron [9]. The drug may have antagonistic effects at the N-methyl-D-aspartate receptor, acting as a non-competitive receptor antagonist—inhibiting the dopamine transporter (DAT) and blocking the removal of DA within the synaptic cleft [10, 11]. Like other neurostimulants, the exact mechanism of amantadine is still unclear and widely debated.

A large, randomized prospective study of minimally responsive traumatic brain injury (TBI) patients in an acute rehabilitation facility showed that taking amantadine twice daily for 2 weeks improves arousal and cognition compared with a placebo [12]. Eight years later, a small retrospective review in *Neurocritical Care* suggests that amantadine, when prescribed as a neurostimulant in hospitalized stroke patients, may help promote more frequent discharges home or to an acute rehabilitation facility versus discharge to a skilled nursing facility or death [11].

Amantadine may therefore prove beneficial as a potential neurostimulant in patients suffering from TBI or acute stroke [11, 12]. However, current American Heart Association/American Stroke Association guidelines do not provide any recommendations for neurostimulants during recovery, most likely due to a lack of randomized controlled trials in this population, and only state that stimulants used to enhance motor recovery are not well established [13]. It still remains unclear whether amantadine is beneficial in improving motor, communicative, cognitive, or behavioral abilities in stroke patients during or after critical care interventions. This lack of guidelines makes it difficult for neurocritical care providers to determine who may benefit from amantadine and when amantadine pharmacotherapy should be initiated during hospitalization. With the current, and limited, amantadine recommendations largely extrapolated from TBI clinical trials, a retrospective analysis was needed in order to assess amantadine utilization and some associations with favorable response in critical care and post-critical care stroke patients.

The primary objective of this study was to assess the differences or associations in demographics data, baseline health characteristics, and different inpatient amantadine dosing regimens between amantadine responders and nonresponders within this small cohort of hospitalized ischemic or hemorrhagic stroke patients at one institution to help inform the design of future randomized controlled trials. The secondary objective was to describe the utilization of amantadine in an institution with a dedicated neurocritical care service that prescribes amantadine to mediate stroke recovery in critical care and post-critical care stroke patients. Given that the American Heart Association/American Stroke Association guidelines do not discuss neurostimulant use to mediate stroke recovery during treatment in critical care or during inpatient rehabilitation, this study can describe amantadine prescription and utilization in recovering hospitalized critical care and post-critical care stroke patients and help inform future prospective clinical trial design [13].

## Methods

### Study design and approval

This was a single-center retrospective study involving critical care and post-critical care stroke patients presenting to an urban level one trauma and comprehensive stroke center with a dedicated neurocritical care service located in downtown Boston, MA. This study received ethics approval by the Tufts Medical Center Institutional Review Board (STUDY00000819) and was conducted in compliance with our institution’s HIPAA guidelines. The Tufts Medical Center Institutional Review Board waived the need for patient consent. The data that support the findings of this study are available from the corresponding author upon reasonable request.

### Cohort description

The overall cohort included all patients ≥ 18 years of age with an acute ischemic or hemorrhagic stroke who presented to an urban comprehensive stroke center between January 1st, 2016, and December 31st, 2019 and were prescribed amantadine hydrochloride for neurostimulation, including wakefulness and language promotion. This cohort only included patients who were admitted to the neurocritical care unit or received consults by the neurocritical care service while admitted to another critical care unit at one point in their admission (*n* = 34). Thus, this sample included only stroke patients that met the criteria to receive neurocritical care during hospitalization and results should not be extrapolated to any other patient populations.

### Data sources

Demographic and hospitalization data were collected from the Tufts Medical Center Neurocritical Care Registry. This database included all patients who were seen by a neurocritical care physician during their hospitalization from January 1st, 2016, to data completion for this study on December 31st, 2019 (*n* = 2,080). Only patients in the database with a diagnosis of ischemic stroke, intracranial hemorrhage, or subarachnoid hemorrhage were included in this study (*n* = 889). Data from this Neurocritical Care Registry were cross-referenced with an institutional stroke registry, when applicable, to ensure the accuracy of data collection. Next, an amantadine prescription list from the Tufts Medical Center pharmacy department database was used to identify patients with these diagnoses who were prescribed amantadine and to collect amantadine-specific data (*n* = 34). Confirmation of amantadine data was performed through a review of physician, nursing, and therapy notes within the electronic medical record. Lastly, data to determine responder status were obtained from physician, nursing, and therapy provider (occupational, physical, and speech therapy) notes within the electronic medical record.

### Variables

Demographic and baseline health variables included age, sex, race, ethnicity, pre-existing health conditions and living situation (Table 1). Stroke characteristics included presenting stroke type (ischemic vs. hemorrhagic), premorbid Modified Rankin Scale (mRS) [14, 15], a widely-utilized, valid, and reliable assessment of the degree of disability after an acute stroke, and admission mRS. Inpatient hospital variables included type of insurance coverage and point of arrival to the critical care unit, including whether the patient was transferred from another institution. Amantadine-specific data included the indication for amantadine prescription (wakefulness vs. aphasia), admitted location upon amantadine initiation, length of hospital stay prior to amantadine initiation, the widely-utilized, valid, and reliable Glasgow Coma Scale (GCS) [16, 17] assessed on the day before amantadine initiation, and the patient’s intubation status when amantadine was first initiated. Amantadine dosing data included starting dose, daily dose at day 9 (last day considered to determine response status), maximum titrated dose, total amantadine administered in the first 9 days (days considered to determine response status), and prescribed outpatient doses (Table 2). Preliminary outcomes data included discharge location, discharge potential as evaluated by the discharging therapists, as well as both hospital discharge mRS scores and outpatient mRS scores.

**Table 1:** Demographics of participants and amantadine responder status

Characteristic	All Recipients	Responders	Non-responders	*p*-value^a^
**Total Sample**	34 (100)	19 (55.9)	15 (44.1)	0.4706
**Type of Stroke**,** n (%)**				
Ischemic	12 (35.3)	7 (36.8)	5 (33.3)	1.0000
Hemorrhagic (ICH, SAH)	22 (64.7)	12 (63.2)	10 (66.7)	
**Age in years**,** Median (IQR)**	65.5 (52.5–74.3)	64.0 (61.0–74.0)	57.0 (63.0–75.0)	0.5432
**Sex**,** n (%)**				
Female	14 (41.2)	7 (36.8)	7 (46.7)	0.7282
Male	20 (58.8)	12 (63.2)	8 (53.3)	
**Race**,** n (%)**				
Asian	8 (23.5)	2 (10.5)	6 (40.0)	0.2639
Black	6 (17.7)	4 (21.1)	2 (13.3)	
White	17 (50.0)	11 (57.9)	6 (40.0)	
Other	3 (8.8)	2 (10.5)	1 (6.7)	
**Ethnicity**,** n (%)**				
Hispanic	1 (3.0)	0 (0.0)	1 (6.7)	0.7649
Non-Hispanic	30 (88.2)	17 (89.5)	13 (86.6)	
Unknown	3 (8.8)	2 (10.5)	1 (6.7)	
**Point of ICU Arrival**,** n (%)**				
Outside Hospital	20 (58.8)	10 (52.6)	10 (66.7)	0.7229
Emergency Department	7 (20.6)	5 (26.3)	2 (13.3)	
Transfer within Hospital	7 (20.6)	4 (21.1)	3 (20.0)	
**Insurance Status**,** n (%)**				
Private	9 (26.4)	5 (26.3)	4 (26.7)	0.1472
Medicare/Medicaid	17 (50.0)	12 (63.2)	5 (33.3)	
Self-pay/Uninsured	1 (3.0)	0 (0.0)	1 (6.7)	
Unknown	7 (20.6)	2 (10.5)	5(33.3)	
**Living Situation**,** n (%)**				
Home with Others	23 (67.6)	11 (57.9)	12 (79.9)	0.1873
Home Alone	7 (20.6)	6 (31.5)	1 (6.7)	
Rehabilitation Facility	1 (3.0)	1 (5.3)	0 (0.0)	
Long-term Care Facility	2 (5.8)	1 (5.3)	1 (6.7)	
Unknown	1 (3.0)	0 (0.0)	1 (6.7)	
**Pre-existing Conditions**,** n (%)**				
Previous Stroke	9 (26.4)	3 (15.8)	6 (40.0)	0.1392
Hypertension	21 (61.8)	11 (57.9)	10 (66.7)	0.7282
Hyperlipidemia	12 (35.3)	6 (31.5)	6 (40.0)	0.7242
Diabetes Mellitus	8 (23.5)	4 (21.1)	4 (26.7)	1.0000
Atrial Fibrillation	6 (17.7)	4 (21.1)	2 (13.3)	0.6722
Coronary Artery Disease	7 (20.6)	3 (15.8)	4 (26.7)	0.6722
Significant Tobacco Use	7 (20.6)	4 (21.1)	3 (20.0)	1.0000
Significant Substance Use	5 (14.7)	3 (15.8)	2 (13.3)	1.0000
**Pre-amantadine GCS**,** Median (IQR)**	10 (8.75–13.25)	12 (9.0–14.0)	9 (9.0-12.2)	**0.0087**
**Pre-morbid mRS**,** Median (IQR)**^**b**^	3.0 (1.0–4.0)	3.0 (1.0–3.0)	3.5 (1.5-4.0)	0.3748
**Admission mRS**,** Median (IQR)**	5.0 (5.0–5.0)	5.0 (4.0–5.0)	5.0 (5.0–5.0)	0.798

^a^Data were analyzed for statistical differences using a 2-tailed Fisher’s Exact Test for dichotomous and categorical variables and Wilcoxon Rank Sum Tests for continuous variables

^b^Pre-morbid mRS *n* = 11 due to limited pre-morbid data from the electronic health record

Abbreviations: ICU = Intensive Care Unit, mRS = Modified Rankin Scale, GCS = Glasgow Coma Scale

**Table 2:** Amantadine hydrochloride prescriptions

Characteristic	All Recipients		Responders		Non-responders		*p*-value
**Reason for Prescription**,** n (%)**							
Wakefulness	29 (85.3)		16 (84.2)		13 (86.7)		1.0000
Verbal Fluency/Aphasia and Wakefulness	5 (14.7)		3 (15.8)		2 (13.3)		
**Location Upon Initiation**,** n (%)**							
Critical Care	12 (35.3)		8 (42.1)		4 (26.7)		0.3490
Intermediate Care	13 (38.2)		8 (42.1)		5 (33.3)		
Medical/Surgical Floor	9 (26.5)		3 (15.8)		6 (40.0)		
**Started While Intubated**,** n (%)**	6 (17.6)		3 (15.8)		3 (20.0)		1.0000
**Inpatient Day Started**, **Median (IQR)**	15 (6.75–22.25)		16 (7–20)		11 (6–32)		0.9033
**Inpatient Doses in mg**, **Median (IQR)**							
Starting Dose	100 (100–200)		100 (100–200)		100 (50–200)		0.2944
Daily Dose Day 9	100 (50–100)		100 (100–100)		100 (50–100)		0.4775
Maximum Dose	100 (100–200)		100 (100–200)		100 (100–200)		0.7924
Total First 9 days	900 (700-1,450)		900 (700-1,700)		900 (600-1,100)		0.7272
**Prescribed at Discharge**,** n (%)**	21 (61.8)		13 (68.4)		8 (53.3)		0.4836
**Outpatient Doses**,** n (%)**							
100 mg	9 (42.9)		6 (46.2)		3 (37.5)		0.2248
200 mg	10 (47.6)		7 (53.8)		3 (37.5)		
400 mg	2 (9.5)		0 (0.0)		2 (25.0)		

Categorical or dichotomous variables are reported as n (%). Continuous data are reported as median (IQR). Data were analyzed for statistical differences using a 2-tailed Fisher’s Exact Test for dichotomous and categorical variables and Wilcoxon Rank Sum Tests for continuous variables

### Determination of responder status

Novel amantadine hydrochloride responder criteria were adopted from Leclerc et al., 2020, a retrospective study published in the journal, *Neurocritical Care* [11, 18, 19]. To date, no widely accepted measures of neurostimulant effectiveness existed for stroke patients. Therefore, Leclerc et al. adapted measures from trials examining amantadine administration in TBI patients, which were based on known pharmacokinetics [11, 18–20]. Following these guidelines, responders were marked by two of the following on a single day within 9 days after the amantadine course was started: [1] An increase in Glasgow Coma Scale (GCS) score ≥ 3 points from pretreatment baseline; [2] Spontaneous clinical improvement in wakefulness or responsiveness documented in neurology physician notes; or [3] Spontaneous clinical improvement in wakefulness or responsiveness documented in physical, occupational, or speech therapy notes [11]. Patients who met the criteria within the 9-day period were placed into the amantadine responder group, while those who did not meet these criteria were placed into the amantadine nonresponder group. Determination of responder status was performed by a blinded member of the study team without visualization of the patient outcomes or possible predictive variables, which helped ensure that responder status would not impact outcome variables in a biased manner. To further help prevent bias in determining responder status, data related to responder criteria were also kept separate from the outcome or predictive data and were not visualized upon determination of responder status. Only the three criteria mentioned above were utilized when determining responder status.

### Statistical analysis

All statistical analysis was performed blinded using STATA 17 (StataCorp. College Station, TX). The relationship between amantadine response status and hospital-specific metrics was evaluated with multivariable regression models, which were adjusted for potential confounders. These potential confounders included all covariates from Table 1 besides premorbid mRS (*n* = 11) due extensive missingness in the dataset and pre-existing health conditions due to the small subgroup sizes and limited variation across responders and nonresponders. Given the retrospective nature and small sample size of this study, demographics data were analyzed for statistical differences using a 2-tailed Fisher’s Exact Test for dichotomous and categorical variables as well as Wilcoxon Rank Sum Tests and Kruskal-Wallis Tests for continuous variables. Nonparametric tests were chosen for analysis, as they are more robust to unequal variances and skewed distributions often found with small samples [21]. Additionally, many categorical variables had 20% of cells with an expected count and average cell count both less than 5 which justified the Fisher’s Exact Test. Continuous data are presented as medians and interquartile ranges (IQR).

## Results

### Overall patient cohort

#### Demographics

The overall cohort included 34 patients: 22 (64.7%) with hemorrhagic strokes and 12 (35.3%) with ischemic strokes. The median age was 65.5 years old (IQR = 52.5–74.3), the majority were male (58.8%), half were white race (50.0%), and the majority were also non-Hispanic (88.2%). Most patients were transferred from an outside hospital to Tufts Medical Center (58.8%), lived at home with others before their stroke (67.6%), and were insured through Medicare/Medicaid (50.0%). Many patients presented with cerebrovascular risk factors including hypertension (61.8%), previous stroke (26.4%), hyperlipidemia (35.3%), diabetes mellitus (23.5%), atrial fibrillation (17.7%), coronary artery disease (20.6%), tobacco use (20.6%), and substance use (14.7%). The median mRS score at admission to Tufts was 5.0 points (IQR = 5.0–5.0) (Table 1). Thirty-one patients (91.2%) were admitted to the dedicated neurocritical care unit and 3 (8.8%) were admitted to the surgical intensive care unit with neurocritical care service consultation.

#### Amantadine prescription

**Fig. 1:**
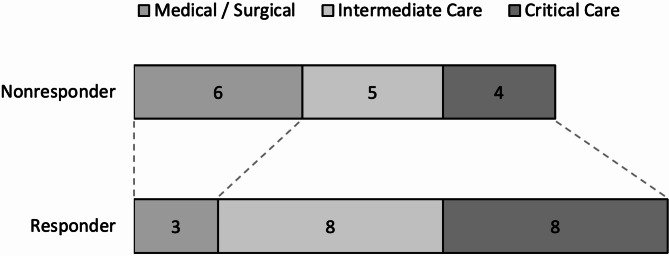
Responder status by amantadine initiation location

Twenty-nine patients (85.3%) received amantadine for wakefulness, while 5 (14.7%) received the drug for both wakefulness and language promotion. Twelve (35.3%) patients were started on amantadine while in critical care, 13 (38.2%) in the step-down unit, and 9 (26.5%) while on a general medical/surgical floor (Fig. 1). The median hospital day number for the first amantadine dose was 15 (IQR = 6.8–22.3). The median starting dose was 100 mg (IQR = 100–200), the dose at day nine of the amantadine course was 100 mg (IQR = 50–100), and the median total milligrams received in the first 9 days of the course was 900 mg (IQR = 700–1,450). The median maximum dose that patients received during their entire hospitalization after dosing adjustments was 100 mg (100–200). At the time of hospital discharge, 21 (61.5%) patients were prescribed amantadine for outpatient continuation. Of these 21 patients, 9 (42.9%) were prescribed 100 mg/day, 10 (47.6%) were prescribed 200 mg/day, and 2 (9.5%) were prescribed 400 mg/day of amantadine in the outpatient setting (Table 2).

### Responder versus nonresponder cohorts

#### Demographics

A total of 19 patients (55.9%) met the criteria for amantadine responder status, while 15 (44.1%) were classified as amantadine nonresponders. Both responders and nonresponders had similar demographic backgrounds (Table 1). Stroke type, age, sex, race, ethnicity, point of arrival, insurance status, living situation, premorbid mRS, and admission mRS did not differ significantly across responders versus nonresponders, although it is worth noting that the true relationships may be underpowered due to the small sample size (Table 1). However, GCS on the day before amantadine initiation was significantly higher for responders (median = 12, IQR = 9–14) than nonresponders (median = 9, IQR = 8–10) using nonparametric tests (*p* = 0.009).

#### Responder criteria

The 19 responders met amantadine responder criteria through various pairings of the three outlined amantadine responder criteria. All 19 (100%) patients were explicitly noted to have spontaneously increased wakefulness on a single day within the physician notes. Seventeen (89.5%) responders met the second criteria through spontaneously increased wakefulness explicitly noted within the physical, occupational, or speech therapy provider notes on a single day, while the 2 other patients (10.5%) met the criteria through a ≥ 3-point increase in GCS from the previous day on a single hospital day, as documented in the nursing provider notes. The median time to responder status was 2 (IQR = 2–4) hospital days and the range was 2 to 9 days.

#### Amantadine prescription

Inpatient doses did not differ significantly between groups, with a median starting dose of 100 mg daily (*p* = 0.294), dose at 9 days of 100 mg daily (*p* = 0.478), and total amantadine within the first 9 days of 900 mg (*p* = 0.727) for both responders and nonresponders. The median maximum dose patients received during their entire hospitalization after dosing adjustments was 100 mg (IQR = 100-200 mg) for both responders and nonresponders, suggesting that there might be no large or significant difference in final increased doses (*p* = 0.792). There was also no significant difference in the outpatient daily doses prescribed between groups (*p* = 0.225) (Table 2).

There was no significant difference in the indication for initiation of amantadine, patient location upon initiation (critical care, step-down unit, general medical/surgical floor), or the number of patients started on amantadine while intubated with univariate statistics, although power should again be considered (Table 2). However, multivariable adjusted models showed a significant association between initiation of amantadine in critical care (versus step-down unit or the general medical/surgical floor) and responder status [𝛃=1.02, 95% CI (0.10, 1.93), *p* = 0.031] (Table 3). These same adjusted models also showed a significant association with initiation in the critical care or step-down unit (versus the general medical/surgical floor) [𝛃=1.06, 95% CI (0.45, 1.85), *p* = 0.002] (Table 4). Among responders, 42.1% were started on amantadine while in the critical care unit and 84.2% of responders were started in the critical care or step-down units combined. Only 15.8% of responders were started on the general medical/surgical floor (Table 2).

**Table 3:** Adjusted model for favorable amantadine response status by initiation in critical care unit versus general medical/surgical floor

Predictor Variable	Crude 𝛃 (95% CI)	*p*-value	Adjusted 𝛃 (95% CI)^a^	*p*-value
Started Critical Care Unit	0.17 (-0.20, 0.54)	0.365	1.02 (0.10, 1.93)	**0.031**
**Demographic and Baseline Health**				
White Race	0.18 (-0.18, 0.53)	0.315	0.02 (-0.69, 0.73)	0.950
Hispanic Ethnicity	-0.67 (-1.66, 0.32)	0.267	-0.28 (-1.49, 0.92)	0.629
Male Sex	0.18 (-0.46, 0.26)	0.577	-0.19 (-1.25, 0.87)	0.717
Age in years	0.00 (-0.02, 0.01)	0.538	-0.01 (-0.03, 0.01)	0.242
Living at Home Alone	0.38 (-0.05, 0.80)	0.079	0.45 (-0.30, 1.21)	0.222
**Stroke Characteristics**				
Hemorrhagic Stroke	-0.04 (-0.41, 0.34)	0.838	0.00 (-1.12, 1.11)	0.995
Admission mRS	-0.24 (-0.52, 0.05)	0.098	-0.16 (-0.54, 0.23)	0.407
**Hospital Metrics**				
Private Insurance	0.00 (-0.41, 0.40)	0.982	-0.19 (-1.14, 0.76)	0.681
Arrived Direct (Not a Transfer)	-0.58 (-1.61, 0.46)	0.424	0.05 (-0.69, 0.79)	0.898
GCS Score before Amantadine	-0.02 (-0.08, 0.05)	0.621	0.03 (-0.04, 0.11)	0.900
Amantadine for Wakefulness Only	-0.05 (-0.55, 0.46)	0.847	-0.04 (-0.93, 0.85)	0.928
Intubated During Amantadine	-0.07 (-0.54, 0.40)	0.758	-0.14 (-0.90, 0.62)	0.698

Adjusted Model R^2^ = 0.40

^a^Adjusted for all covariates in model

Abbreviations: Confidence Interval = 95% CI, mRS = Modified Rankin Scale, GCS = Glasgow Coma Scale

**Table 4:** Adjusted model for favorable amantadine response status by initiation in critical care or step-down unit versus general medical/surgical floor

Predictor Variable	Crude 𝛃 (95% CI)	*p*-value	Adjusted 𝛃 (95% CI)^a^	*p*-value
Started Critical Care/Step-Down Unit	0.31 (-0.08, 0.70)	0.119	1.06 (0.45, 1.68)	**0.002**
**Demographic and Baseline Health**				
White Race	0.18 (-0.18, 0.53)	0.315	0.34 (-0.26, 0.95)	0.254
Hispanic Ethnicity	-0.67 (-1.66, 0.32)	0.267	-0.20 (-1.23, 0.84)	0.696
Male Sex	0.18 (-0.46, 0.26)	0.577	-0.21 (-1.13, 0.70)	0.631
Age in years	0.00 (-0.02, 0.01)	0.538	-0.01 (-0.03, 0.00)	0.116
Living at Home Alone	0.38 (-0.05, 0.80)	0.079	-0.08 (-0.78, 0.63)	0.819
**Stroke Characteristics**				
Hemorrhagic Stroke	-0.04 (-0.41, 0.34)	0.838	-0.50 (-1.34, 0.33)	0.223
Admission mRS	-0.24 (-0.52, 0.05)	0.098	-0.26 (-0.60, 0.08)	0.124
**Hospital Metrics**				
Private Insurance	0.00 (-0.41, 0.40)	0.982	0.24 (-0.54, 1.02)	0.525
Arrived Direct (Not a Transfer)	-0.58 (-1.61, 0.46)	0.424	0.37 (-0.31, 1.05)	0.268
GCS Score before Amantadine	-0.02 (-0.08, 0.05)	0.621	0.00 (-0.07, 0.06)	0.875
Amantadine for Wakefulness Only	-0.05 (-0.55, 0.46)	0.847	-0.62 (-1.43, 0.18)	0.124
Intubated During Amantadine	-0.07 (-0.54, 0.40)	0.758	-0.19 (-0.84, 0.47)	0.556

Adjusted Model R^2^ = 0.55

^a^Adjusted for all covariates in model

Abbreviations: Confidence Interval = 95% CI, mRS = Modified Rankin Scale, GCS = Glasgow Coma Scale

When examining the day of initiation, the median hospital day number for the first amantadine dose was 16 (IQR = 7–20) for responders and 11 (IQR = 6–32) for nonresponders, which presents no significant difference (*p* = 0.903). Additionally, adjusted models found no significant association between the hospital day number started and responder status [𝛃=-0.003, 95% CI (-0.02, 0.02), *p* = 0.772]. Kruskal-Wallis tests suggest that the median hospital day number in which amantadine was initiated was not significantly different between starting locations (*p* = 0.5467). The median hospital day number on which amantadine was initiated was 17 days for patients in the critical care unit (IQR = 5-20.75), 11 days in the step-down unit (IQR = 8-16.5), and 19 days in the general medical/surgical floor (IQR = 6.5–47.5). Hospital day number initiated did have more variance in the general medical/surgical floor compared to the critical care unit or step-down unit, which again needs to be examined in a larger sample. However, models adjusted for both hospital day number and location of initiation also showed a significant association between initiation of amantadine in critical care (versus step-down unit or the general medical/surgical floor) [𝛃=1.01, 95% CI (0.07, 1.96), *p* = 0.036] and initiation in the critical care or step-down unit (versus the general medical/surgical floor) [𝛃=1.11, 95% CI (0.48, 1.74), *p* = 0.0016] and responder status. Crude regression models showed no significant association between amantadine initiation on an earlier hospital day number and fewer days until meeting the criteria to become an amantadine responder [𝛃=0.02, 95% CI (-0.10, 0.05), *p* = 0.5281].

#### Amantadine termination

While no amantadine-specific side effects were noted within the electronic medical record, amantadine was stopped in several patients before hospital discharge for other reasons. One patient was diagnosed with neutropenia and leukopenia, so amantadine was stopped on day 5, but this patient had already met the responder criteria. Amantadine was initially held in another patient who had increased liver function tests, but it was later restarted and this patient met nonresponder criteria. Additionally, one patient had an acute respiratory event from a mucous plug and was re-intubated, so amantadine was stopped on day 9, but this patient already met responder status. Lastly, amantadine was discontinued in one patient after emergency surgical evacuation of a recurrent spontaneous intraparenchymal hemorrhage, but this patient already met nonresponder criteria before their death. While these are not known side effects of amantadine use, reporting these events is important to understand the medication course within this cohort.

### Outcomes

Although this small, retrospective study was not powered or designed to definitively examine associations between amantadine response status and clinical outcomes, we wanted to describe associations in order to examine metrics to determine if they should be considered for inclusion in future randomized controlled trials. Regarding discharge location, amantadine responders were positively associated with discharge to an acute rehabilitation facility [𝛃=0.41, 95%CI (-0.06, 0.89), *p* = 0.086], although this association was not statistically significant, which should be interpreted sparingly due to the small sample size. A total of 14 responders versus 3 nonresponders were discharged to an acute rehabilitation facility, which may suggest clinical significance. No patients within this study were discharged home. While actual discharge to an acute rehabilitation facility was statistically nonsignificant through multivariable analysis, amantadine responders were significantly positively associated with a higher *potential* for acute rehabilitation placement as evaluated by discharging therapists [𝛃=0.58, 95% CI (0.24, 0.91), *p* = 0.002]. After initiation, amantadine responders were associated with hospital discharge 15 days earlier than nonresponders, although this was found to be statistically nonsignificant, and again should be interpreted sparingly [𝛃=-15.19758, 95% CI (-42.59, 12.20), *p* = 0.258]. Additionally, the median time from amantadine day 9 to hospital discharge for all recipients was 3.5 days. After day 9, amantadine responders were associated with hospital discharge 14 days earlier than nonresponders, although this was found to be statistically nonsignificant and should not be interpreted as a definitive conclusion due to the small sample [𝛃=-14.06, 95% CI (-40.21, 12.08), *p* = 0.2722]. While the median discharge mRS score was 5 for both responders and nonresponders, discharge mRS score was significantly negatively associated with amantadine responder status [𝛃=-0.48, 95% CI (-0.92, -0.03), *p* = 0.037] at the time of hospital discharge. Additionally, while median outpatient mRS score was 4 for both responders and nonresponders, outpatient mRS score was negatively associated with amantadine responder status [𝛃=-2.02, 95% CI (-5.07, 1.04), *p* = 0.127], although this relationship was not found to be statistically significant.

## Discussion

This study evaluated factors associated with favorable amantadine responder status among hospitalized current or post-critical care stroke patients with decreased level of consciousness to inform the design of future randomized controlled trials. The examination included nonparametric and multivariable models adjusted for potential confounding and a cohort representative of the racial demographics of the United States [22]. While we are explicit to suggest that the sample size may have led to some underpowered statistical analysis, results suggest that there may not be many significant differences or associations in measured predictive variables between amantadine responders and nonresponders, other than GCS which may be higher for responders on the day before amantadine initiation. There were also large variations in amantadine dosing regimens across patients in this study. Most importantly, amantadine initiation during higher acuity in the critical care or step-down units may be related to favorable responder status within this small cohort of hospitalized ischemic and hemorrhagic stroke patients, however, studies with larger sample sizes are needed to validate preliminary findings.

### Demographics

Nonparametric univariate tests showed no statistically significant differences in most demographic characteristics between groups. For example, nonresponders were not significantly older, did not have more pre-existing conditions, and did not have a particular stroke type. Additionally, there was no significant difference in pre-morbid or admission mRS, which suggests that there may have been similar disability rates before stroke or upon admission between the two cohorts. However, GCS on the day before amantadine initiation was significantly higher for responders compared to nonresponders, which suggests that increased levels of consciousness directly before starting amantadine may be related to increased amantadine response. Despite this, the median GCS for both groups was below the normal score of 14 or 15. Given these findings, a large randomized controlled trial should examine amantadine across different levels of GCS to determine if there is a threshold of GCS at which there is less benefit of amantadine initiation. Even though we found that patients with higher GCS might be more likely to respond to amantadine when compared to patients with lower GCS, a clinical trial is needed to examine clinical-effectiveness thresholds and guidelines regarding initiation in patients with mid-to-lower GCS scores. Similarities between groups except for higher GCS for responders may suggest that there may be limited significant crude relationships between these possible predictive variables and favorable responder status, however, the small sample size limits definitive conclusions. While a randomized trial would balance demographic factors, our study suggests that there could be limited baseline differences between cohorts.

A previous retrospective investigation of amantadine in hospitalized stroke patients found similar demographic characteristics to our study cohort, including a median age of 66 years old, 61% male, 66% hemorrhagic stroke, and 34% ischemic stroke, compared to the median age of 66 years, 59% male, 65% hemorrhagic stroke, and 35% ischemic stroke within our study [11]. This suggests that these demographics might represent the average for patients started on amantadine in this setting. While 96% of amantadine recipients in the prior study were White, *50.0% of patients from our study were from non-White racial backgrounds* [11]. Our cohort comprises 50.0% White, 23.5% Asian, 17.7% Black, and 8.8% individuals from other races. This may suggest that our study results could be more generalizable to the current United States population, which included 76.3% White, 5.9% Asian, and 13.4% Black individuals as of 2019 [22].

### Amantadine hydrochloride prescriptions

Since the American Heart Association/American Stroke Association does not discuss neurostimulant administration in their guidelines, there are no well-accepted recommendations regarding amantadine dosing quantities or regimens [13]. The only recommendations for amantadine usage come from prior clinical trials, usually examining TBIs, which are also not consistent in dosing [12, 23]. Given this absence in guidelines, patients within this current study were started on different doses of amantadine depending on the provider, ranging from 50 mg to 200 mg daily, with an average of 100 mg. Despite these variations, the analysis showed no significant difference in amantadine doses at initiation, day 9, or maximum dose between groups, although definitive conclusions cannot be made due to the small sample size within this study.

Clinically, doses seem to differ at our institution based on different care teams referencing separate TBI clinical trials. However, basic science studies in rodents have shown that higher doses of amantadine (40 mg/kg) are not more efficacious in promoting neurological recovery in TBI rodent models when compared to smaller doses (20 mg/kg) [24]. This non-dose-dependent phenomenon may result from negative feedback mechanisms within the nervous system, where presynaptic receptors decrease DA release into the synapse and postsynaptic neurons begin to downregulate their binding of DA, which limits the physiological effects of increased titration of amantadine [25–27]. Weight-based doses of amantadine are also much higher in these rodent studies (10–40 mg/kg) compared to human trials (often 50–200 mg/dose total or up to 2.50 mg/kg for the average weight adult), which may suggest possible underdosing in human stroke patients which is not explored here and needs further investigation in subsequent studies [11, 12, 23, 24].

The analysis also showed no association between responder status and amantadine initiation on an earlier hospital day number, which may suggest that earlier dosing may not be more beneficial. Similar results were found in a previous randomized controlled pilot trial of amantadine within TBI patients, which showed that earlier treatment with amantadine was not more efficacious in improving functional or cognitive outcomes compared to later treatment [23]. Additionally, amantadine was initiated at least 8 days earlier, on average, by Leclerc et al. in their retrospective study, but the percentage of hospitalized stroke patients who met responder status in that study was similar to our study at 56% versus 53% [11]. This may help suggest that earlier initiation of amantadine based on hospital day number may not be associated with higher rates of responders. Again, this warrants further examination. Lastly, a trial of amantadine in hospitalized critical care patients with non-traumatic brain injury showed that early initiation within 3 days of admission on average did not have significantly higher odds of increased consciousness compared to late initiation within 13 days [28]. However, this study found that those with delayed initiation (average 8 days) did have significantly higher odds of increased consciousness [28]. Together, these findings along with our results may suggest that a favorable increase in consciousness may not be related to an earlier hospital day number of amantadine initiation.

Although there were no associations between responder status and amantadine initiation on an earlier hospital day number in this current, small study or the existing literature, our multivariable models adjusting for baseline demographic, health, and hospital covariates found that amantadine administration during higher acuity in critical care or step-down units may be associated with an increased favorable response status in our study. While univariate statistics showed no significant difference, twice the number of responders were started on amantadine in both the critical care and step-down units when compared to the general medical/surgical floor, which may suggest clinical significance regarding acuity of initiation. This discrepancy may be due to insufficient power for the analysis and not true non-significance. It is also possible that providers in these critical care settings may have higher comfort prescribing amantadine, which could be examined quantitatively and qualitatively in subsequent studies. Additionally, analysis showed that the median hospital day number initiated was not significantly different between starting locations, which suggests that patients in higher acuity settings (critical care or step-down units) may not actually be receiving amantadine significantly earlier than patients of lower acuity (floor). The existing literature examining amantadine use in hospitalized stroke patients based on acuity is extremely limited, however, a recent systematic review suggests that amantadine initiation in the critical care unit may increase post-stroke recovery. Specifically, amantadine started in critical care post-stroke patients showed improvements on their Coma Recovery Scale and Disability Rating Scale on day 5 after initiation [29, 30]. This scale was designed by clinicians in 1991 and revised through multiple iterations to develop a standardized approach for assessing disorders of consciousness and was found to be both reliable and valid in subsequent studies [31–33]. In contrast, studies examining later post-stroke amantadine in mostly non-acute hospitals or rehabilitation facilities found that only 2 of the 4 studies had overall improvement with amantadine, including higher word-finding, as well as higher activity and cognitive, emotional, and motor function [30, 34, 35].

While patients can be at various stages of their recovery on any given hospital day number, this method of analysis considers the acuity of the patient’s condition in relation to amantadine response status. In the retrospective study by Leclerc et al., all 79 patients had amantadine started during critical care and the authors found an overall amantadine response rate of 53%, while this current study found that 8 of the 12 patients (67%) started in the critical care unit were classified as amantadine responders [11]. In contrast, only 3 of the 9 patients (33%) who started on the general medical/surgical floor within our study were classified as amantadine responders. While our current sample size is too small to make any clinical recommendations or definitive conclusions, Leclerc et al. mention that the optimal time to initiate neurostimulants after acute stroke is still unknown, and voiced caution in very early amantadine initiation as some studies suggest that very early mobilization within 24 h of a stroke may not be associated with better recovery [30, 36, 37]. Together, this could suggest that favorable response status may be more related to the patient’s *level of acuity* and not earlier initiation based on hospital day. We need to examine if there may be an optimal time for initiation based on the patient’s current health status.

This current study also examined the timeline from amantadine initiation to responder status and found a median time of 2 days (IQR = 2–4; range 2–9 days), which was consistent with the previous study that found a median response of 3 days (IQR = 2–5; range 1–9 days) [11]. Another small retrospective study of patients with only hemorrhagic stroke found an average response time of 3 to 7 days after initiation [38]. These timelines all align with known amantadine pharmacokinetics of a maximum plasmatic half-life of approximately 2 days and a steady-state response of 4 to 7 days in Parkinsonian patients, which may also be representative of stroke patients [20]. Similarities in both the percentage of responders and median time to response status between these inter-institutional studies may help to suggest *validated timelines* for providers to determine responder status and determine future care plans for these patients.

For patients who cannot return home post-stroke due to residual deficits, the preferred post-discharge placement is an acute inpatient rehabilitation facility, where patients participate in intensive cognitive, language, and physical therapy sessions up to three hours per day [39]. However, external factors such as socioeconomic status, race, and insurance status have been found to cause disparities in rehabilitation placement in patients who would have otherwise received benefit from discharge to an acute rehabilitation facility [40]. For this reason, this study also examined the potential for rehabilitation placement as evaluated by discharging therapists. When examined together, positive associations between responder status and both acute rehabilitation facility placement and high recommendation for rehabilitation placement show that randomized controlled trials could examine the relationship between amantadine and discharge outcomes. Previous retrospective examination of amantadine in hospitalized stroke patients in the journal *Neurocritical Care* found that 90% of responders versus 62% of nonresponders were discharged to an acute rehabilitation facility, while our study showed similar trends with 74% and 20%, respectively [11]. Additionally, amantadine responders were found to be discharged approximately two weeks earlier than nonresponders after drug initiation. Although this value was not found to be statistically significant, this trend for earlier discharge paired with more frequent discharges to acute rehabilitation facilities in responders suggests that future trials could also examine the associations between amantadine initiation and time to hospital discharge.

### Limitations

This study was limited by its retrospective nature. An initial retrospective review was necessary to evaluate possible associations and investigate the practicality of future clinical investigation. Since the data within this study were collected through chart review, there exists a limitation in human collection errors. To reduce differences between research assistants, variable definitions were standardized, and study members all received the same training regarding data collection methods. All individual variables were collected by a single study member, which ensured consistency in classification. Additionally, demographics data and mRS were cross-referenced with an institutional stroke registry, when applicable, to ensure the accuracy of data collection.

The study was also limited by a relatively small sample size at only one academic institution. Over the 4-year period of this retrospective evaluation from the neurocritical care cohort, only 34 patients were eligible for analysis. To attenuate the potential effects of this limitation, statistical analysis was performed using both univariate nonparametric models and multivariate models, which factored in potentially confounding variables. These nonparametric tests are more robust to unequal variances and skewed distributions often found with small samples [21]. In some cases, this small sample size may have been too low to drive statistical power, which might have possibly been avoided with a larger cohort [21]. Nonsignificant findings in this study may be attributed to low statistical power and not actual nonsignificance, so further analysis with a larger sample size would be needed to prove this theory. Therefore, these results cannot conclude that associations or differences do not truly exist and can only suggest that there is not enough evidence to make a determination. We cannot make any clinical recommendations with this data and can only suggest what additional subsequent trials should possibly include in their study.

Additionally, the small sample sizes contributed to some large confidence intervals surrounding the coefficients and therefore less certainty regarding the true estimate. Given this limitation, this study only examined the presence of significant associations and did not attempt to exponentiate these values into odds ratios, which would falsely overestimate the effect size [41]. While this study suggests that there may be some associations between measured predictive factors and amantadine response status, we believe that it does not have the power to accurately report effect sizes through odds or risk ratios, so these values are not reported [41]. Additionally, there may be other predictive factors that were not measured in this study that could impact the results. Overall, we are conservative with our interpretations and cannot make definitive conclusions.

Lastly, a major limitation of this study is that patient acuity may actually be affecting the determination of response status and falsely skewing favorable results toward administration in critical care or step-down units. Patients started on amantadine in the critical care or step-down units are more acute and may be receiving more regular comprehensive assessments by the providers which could increase the detection of improvement in consciousness and lead to classification as an amantadine responder. Given that 100% of patients met responder criteria through physician notes and 89.5% met the second criteria through physical, occupational, or speech therapy provider notes, these subjective methods may be introducing bias. However, these are currently the only published guidelines to determine the amantadine response status of hospitalized stroke patients. Additionally, neurocritical care physicians in higher acuity settings may be more familiar with amantadine protocols and more likely to prescribe it. Similarly, there exists a chance that patients who are started on amantadine in one unit, such as critical care, but are transferred to another unit (step-down or floor) may be missed for criteria as different providers may not be looking for this change in consciousness as closely as the initial prescribing physician. However, this may be limited since an objective measure of increase in GCS score ≥ 3 points from pretreatment baseline was also used in the daily determination of responder status.

### Future design suggestions

While existing literature is rich in randomized placebo-controlled trials of amantadine in TBI patients, there are no published randomized trials with critical care stroke patients [12, 18, 23]. Given the utilization of amantadine in hospitalized stroke patients across numerous institutions, future studies must include randomized controlled trials of amantadine in this population. We identified some key variables that may be included within these future study designs: [1] Given the large variation in dosing regimens identified within this study and other prior studies, the trials could examine any dose-dependent relationships with clinical outcomes to establish stroke-specific dosing guidelines [2]. We identified that GCS at the time of amantadine initiation could be examined as a predictor of clinical outcomes and future trials could work to identify a minimum GCS threshold for which amantadine initiation is still efficacious [3]. Finally, our study suggests that clinical trials could also examine the impact of patients’ *level of acuity* on clinical outcomes in addition to earlier amantadine initiation based on hospital day number.

## Conclusions

Retrospective univariate analyses suggest that there may not be many significant differences in most measured predictive variables, including demographics data, baseline health characteristics, or different inpatient amantadine dosing regimens between amantadine responders and nonresponders within this small cohort of hospitalized ischemic and hemorrhagic stroke patients. However, while nonsignificant, large variations in amantadine dosing regimens were identified which suggests the need to establish stroke-specific dosing guidelines for critical care and post-critical care stroke patients. Additionally, associations with GCS and favorable amantadine response status may suggest the need to further identify a minimum GCS threshold for which amantadine initiation is still efficacious which can be included within amantadine initiation guidelines. Lastly, initiation of amantadine during *higher acuity* settings in the critical care or step-down units may be related to favorable responder status. This suggests that it may be important to consider the patient’s *level of acuity* when considering amantadine to help mediate decreased level of consciousness in hospitalized stroke patients, instead of solely examining earlier initiation based on hospital day number. Overall, we suggest that future randomized controlled trials could particularly examine the impact of various dosing regimens, GCS thresholds, and patients’ *level of acuity* during amantadine initiation on clinical outcomes.

## Data Availability

The data that support the findings of this study are available (anonymized) from the corresponding author upon reasonable request of any qualified investigator for purposes of replicating procedures and results.

## Abbreviations

TBI: Traumatic brain injury
mRS: Modified Rankin Scale
GCS: Glasgow Coma Scale
IQR: Interquartile ranges
